# The gut microbiome in early pregnancy is associated with the severity of nausea and vomiting: a nested case‒control study

**DOI:** 10.1080/29933935.2025.2603861

**Published:** 2025-12-22

**Authors:** Clàudia González-Valdivia, Bangzhuo Tong, Sanna Hjalmarsson, Unnur Guðnadóttir, Nicole Wagner, Lars Engstrand, Ina Schuppe-Koistinen, Emma Fransson, Stefanie Prast-Nielsen, Nele Brusselaers, Luisa W. Hugerth

**Affiliations:** a Department of Marine Biology and Oceanography, Institut de Ciències del Mar, Barcelona, Spain; b Department of Medical Biochemistry and Microbiology, Science for Life Laboratory, Uppsala University, Uppsala, Sweden; c Unilabs Sverige, Stockholm, Sweden; d Department of Women's and Children's Health, Karolinska Institute, Solna, Sweden; e Department of Medicine, Karolinska Institute, Solna, Sweden; f Department of Molecular, Tumour and Cell Biology, Karolinska Institute, Solna, Sweden; g Department of Women’s and Children’s Health, Uppsala University, Uppsala, Sweden; h Department of Clinical Neurosciences, Karolinska Institute, Solna, Sweden; i Global Health Institute, University of Antwerp, Antwerp, Belgium

**Keywords:** Gut microbiome, nausea, vomiting, pregnancy, nested case–control, population-based

## Abstract

Approximately 70% of all pregnancies are affected by nausea and vomiting (NVP), yet the mechanisms controlling this phenomenon are not well known. Pregnancy hormones explain a large part of this effect, mostly through human chorionic gonadotropin and fetal production of GDF15, a hormone active in the brain stem. Still, there is a wide variation in the severity of symptoms, ranging from no nausea to severe vomiting requiring hospitalization (hyperemesis gravidarum). Here, we present a nested case‒control study within the large SweMaMi cohort, wherein 337 participants with severe NVP in early pregnancy were matched 1-to-1 with moderate and mild NVP, respectively. Subjects with more severe nausea had lower richness and diversity in their fecal microbiomes. Several taxa were significantly associated with NVP score, where the most extreme are a negative correlation with Lactobacillaceae and positive correlations with Bifidobacterium dentium and Puniceicoccaceae. Finally, higher NVP score was associated with a higher abundance of bacteria encoding for the neuroactive pathways of glutamine degradation, inositol synthesis, and lactate production. In conclusion, the gut microbiota was strongly associated with NVP. Further studies with direct interventions capable of restoring the early-pregnancy gut microbiome could open up new approaches for dealing with the most common symptom of early pregnancy.

## Introduction

Nausea and vomiting of pregnancy (NVP) are experienced by roughly 70% of pregnant individuals.^1^ Symptoms of nausea and/or vomiting can start as early as gestational week 4 and usually peak at week 8–16. In 95% of pregnancies, NVP has ceased by week 22, but some individuals retain symptoms into the third trimester.^2^ NVP is associated with negative outcomes for pregnant people with higher rates of hypertension, preeclampsia,^3^ as well as lower quality of life.^4^ Paradoxically, NVP is associated with lower likelihood of miscarriage, preterm delivery and low birthweight,^3^^,^^5^ as well as a lower risk for birth defects.^6^ These observations have led to two alternative, but not-exclusive explanations for NVP: that it is an evolutionary adaptation meant to protect the fetus from teratogens^7^^,^^8^ and that it is a side-effect of the metabolism of a viable fetus. The latter observation was initially supported by a direct correlation between human chorionic gonadotropin (hCG) levels and nausea symptoms,^9^^,^^10^ and has recently been strengthened by evidence that fetal production of GDF15, a hormone active in the brain stem, plays a causative role in NVP.^11^ An extreme form of NVP is hyperemesis gravidarum (HG) which affects up to 3% of pregnant individuals^12^^,^^13^ and requires hospitalization and fluid replacement therapy. While NVP is protective for the fetus, HG is associated with worse outcomes both for the pregnant person and the fetus. HG can lead to weight loss, liver dysfunction, renal failure, and depression.^14^^,^^15^ Children born from people with HG have higher rates of low birthweight, being small for gestational age, and preterm delivery.^16^

Several risk factors have been established for NVP and HG, including sociodemographics, characteristics of the pregnancy, and medical history. Genetics have been shown to play a part through several studies of NVP and HG,^17^^,^^18^ as well as a lower pre-pregnancy level of GDF15.^11^ The risk of developing severe NVP/HG is increased with prior miscarriages, multiple gestation, Down’s syndrome and molar pregnancies.^12^^,^^13^^,^^19^ In addition to this, HG is also associated with HG during a previous pregnancy, female sex of the fetus and being underweight, overweight, or obese.^19^^,^^20^ Previous experience of nausea from motion sickness or migraines is also associated with a higher incidence of NVP/HG, and there is a higher likelihood of NVP/HG if close family members have experienced any of the conditions.^21^ Higher age and smoking are associated with lower risk of both NVP/HG and other kinds of nausea (such as post-operative or chemotherapy-induced).^22‐24^ Common medications recommended for mild to moderate NVP in Sweden include proton-pump inhibitors (PPI) and histamine H1 receptor antagonists (H1RA).^25^ Previous research shows that PPI has a strong effect on gut microbiome composition, which is not observed for histamine antagonists (H1RA or H2RA).^26^

Emerging evidence suggests that alterations in the intestinal microbiome may contribute to the pathophysiology of hyperemesis gravidarum (HG). Women with HG have been reported to harbor distinct microbial profiles compared to asymptomatic pregnancies, including higher *α*-diversity and enrichment of taxa within Bacteroidaceae, Firmicutes, Clostridia, and Betaproteobacteria lineages.^27^ Specific bacterial groups, such as Defluviitaleaceae UCG-011, *Ruminococcus spp.*, *Turicibacter*, and members of the phylum Verrucomicrobiota, have been positively associated with HG risk, whereas *Coprococcus2* appears to be inversely associated.^28^ In parallel, women with HG have twice the prevalence of *Helicobacter pylori* infection compared to other pregnant persons, and women with *H. pylori* have a 3-fold increased risk of developing HG.^29^

Despite these findings, prior investigations have largely relied on 16S rRNA gene sequencing or real-time PCR, limiting their capacity to resolve species-level taxonomic annotation and microbial function. In contrast, by leveraging shotgun metagenomic sequencing, our study enables functional annotation of the gut microbiome, providing new insight into the metabolic pathways that may mediate gut–brain communication underlying nausea and vomiting in pregnancy. Moreover, rather than a binary comparison between HG and non-HG pregnancies, we investigated whether variation in the fecal microbiome composition could partially explain the severity continuum of NVP associated with physiological changes during pregnancy.

## Materials and methods

### Participant recruitment and variables

This study is nested within a large prospective cohort study, the Swedish Maternal Microbiome (SweMaMi) project, which recruited 5439 participants all over Sweden between 2017 and 2021.^30^ The Regional board of Ethics, Stockholm, Sweden has approved the study protocol (2017/1118-31), which was performed according to the declaration of Helsinki. Participants were informed of the study and gave consent through the study webpage before filling out the first questionnaire.^30^ Consent could and can be withdrawn at any time by contacting the study team. The questionnaires contained questions on demographic, health, and lifestyle characteristics such as marital status and smoking habits, as well as standardized questions for mental distress (Edinburgh Postnatal Depression Scale, EPDS),^31^ stress (Cohen Perceived Stress Scale 4, PSS-4),^32^ and nausea (24-hour pregnancy-unique quantification of emesis (PUQE))^33^ and the Bristol Stool Form Scale.^34^ A poor diet score (range 0–5) reflects overall dietary quality, with higher scores indicating a less healthy diet. One point was assigned for each response other than “daily” to questions about fruit, vegetable, and whole grain bread consumption. In addition, responses of “daily” or “several times weekly” to questions about sugar and sweetened beverage intake contributed one point each. The socioeconomic score (range 0–3) was used to capture socioeconomic status, with one point given for each of the following: having a bachelor's degree or higher, being in a relationship, and working full time.

In this work, we analyzed data from the first questionnaire, filled in during weeks 8–16 of pregnancy, and the first fecal sample, sent in within two weeks of the questionnaire. Exclusion criteria were use of antibiotics during the pregnancy or in the previous three months, and multiple gestation. Anyone who had not completed the questions used to calculate the PUQE score was also excluded. After filtering for these criteria 3990 subjects remained. Of these, for logistic reasons, 1254 had available data at the start of this study.

### Case‒control selection

Participants with fecal samples with a total read count of <20 M reads after human DNA removal were excluded, leaving 1239 subjects. Participants with PUQE scores between 3 and 6 were considered to have mild NVP, while those with high PUQE score (PUQE ≥ 7, *N* = 551) were classified as having moderate to severe NVP, consistent with the thresholds defined in the original PUQE validation study.^33^ To account for variation within the mild NVP range and avoid obscuring microbial differences when matching cases with controls of differing symptom levels, we further stratified mild NVP subjects into two groups: low PUQE score (PUQE 3–4, *N* = 349) and medium PUQE score (PUQE 5–6, *N* = 339). Propensity score matching was done with the “matchit” function from the MatchIt v4.5.5 R package^35^ on age (y), body mass index (BMI, kg/m^2^), parity (nulliparous or not), and week of gestation at sampling, the latter included to minimize the effect of individual variability related to gestational timing. Neighbor joining and optimal pair matching were evaluated. Optimal pair matching was chosen as there was less distance (distance = “glm”, link = “probit”). After excluding 2 medium-PUQE and 1 low-PUQE participant with missing information on matching variables, each high-PUQE case was matched with one low-PUQE and one medium-PUQE control, resulting in a total of 337 matched triplets (1011 participants) included in the final analysis.

### Fecal sample collection, extraction, sequencing, and annotation

Fecal samples were collected by participants using a DNA/RNA Shield-Fecal collection tube (Zymo Research, California, USA) from toilet paper with the spoon attached to the lid of the collection tube. Samples were mechanically lysed in FastPrep-24 before beads were removed and samples were purified. Library preparation was performed with a brief PCR amplification step in an MGISP-960. The 850–880 bp fragments were selected using the electrophoresis-based size selection with a 4200 TapeStation. The fragments were then normalized to the same concentration and circularized. The DNA nanoballs (DNB) were loaded into array flow cells with the DNB Loader MGIDL-T7. Sequencing was performed using MGI sequencers (MGI Tech Co., G400 or T7 models) on paired 150-base pair reads.

For read processing, we used the StaG-mwc workflow^36^ based on Snakemake.^37^ This workflow uses FastP^38^ for trimming of adapter sequences and low-quality bases. Host reads were then removed through mapping to the human reference genome GRCh38 with Kraken 2.^39^ Finally, the sequences were annotated with MetaPhlAn4.0^40^ for taxonomic annotation and HUMAnN3^41^ for functional annotation. Gene-families annotated from HUMAnN3 were further converted into KEGG and EggNOG functional groups using the regroup function from the HUMAnN3 package, and eventually grouped into profiles of gut–brain modules^42^ (GBM) and gut–metabolic modules^43^ (GMM) with the Omixer-RPM.^44^ Species-level tables are available as Table S1, and gut modules in Tables S2 and S3.

### Subject-level variables

Participant-level variables are described in Table S4. These were mostly based on answers to the first questionnaire which was filled in during pregnancy week 8–16, except medication usage is supplemented with data from the Swedish National Health Records, considering prescriptions made up to three months before sampling, but no more than one week.

### Statistics

Statistical analyzes and plotting were done using R Statistical Software v4.3.1. The data were cleaned using packages from the tidyverse v2.0.0^45^ and plotted with the ggplot2 package v3.4.4.^46^

#### Covariable analysis

To test for correlation between subject-level variables and NVP severity, χ^2^ test was used for categorical variables and the Kruskal‒Wallis test was used for quantitative variables. Pairwise comparison between groups were made with Wilcoxon’s rank sum test.

Correlations between the PUQE score and subject-level variables were analyzed with Spearman's correlation. Correlations between NVP severity categories and other variables were tested with the χ^2^ test for qualitative variables and the Kruskal‒Wallis test for quantitative variables. Pairwise comparisons between groups were conducted using Wilcoxon's rank sum test. The variables that significantly correlated with the PUQE score or NVP severity were kept as potential confounders in further analysis.

#### Within-sample diversity

Diversity metrics were calculated using the vegan package v2.6.4.^47^ Species that appeared in less than 8 samples were removed. Alpha-diversity was calculated as Shannon's entropy, Pielou's evenness and observed species. For calculation of the observed species, samples were rarefied to the sampling depth of the sample with the lowest read count (*N* = 23,209,862) using the function “rarefy”. Reads after host removal were used to convert the relative abundances from the MetaPhlAn4^40^ output to abundances to use for rarefaction. Correlations between alpha-diversity metrics and quantitative variables were calculated using Spearman's correlation. Correlations between alpha-diversity metrics and qualitative variables were calculated with Kruskal‒Wallis tests.

#### Between-sample distance

The “vegdist” function was used to calculate beta-diversity as Jaccard dissimilarity (method = “jaccard”, binary = TRUE) and Aitchison distance (method = “robust.aitchison”). The Aitchison distance was calculated as a robust Aitchison which only CLR-transformed values above zero. To calculate significance levels a permutational multivariable analysis of variance (PERMANOVA) was conducted using the function “adonis2” (na.action = na.omit, permutations = 9999). The PUQE score, NVP severity and potential confounders were used as independent variables. Univariable analysis was conducted for each of the variables and a multivariable model was constructed with variables that were significant in the univariable model with either the PUQE score or NVP severity in each model.

#### Analysis of abundance of species

An analysis of the abundance of species was done using the “ancombc2” function from the ANCOMBC package v2.4.0.^48^ Variables were chosen based on the results from the beta-diversity. Variables that were significant in the multivariable model from PERMANOVA and could not be explained by other variables were used. The models used quantitative variables as fixed effects.

#### Gut-brain modules

To account for the abundances of microbial genes not mapped to gut–brain modules (GBMs) or gut-metabolic modules (GMMs), an additional feature labeled “Others” was introduced for each sample to guarantee a total sum of 1 million and quantification of features as counts per million (CPM). Modules whose prevalence was <15% were excluded from the analysis. Those with prevalence >15% and ≤85% prevalence were analyzed in terms of presence–absence using a logistic regression on PUQE scores. Depression, stress, eating disorders, and diet were included as covariables. For modules with a prevalence exceeding 85%, partial Spearman's rank correlation was used. Both the logistic regression and the linear regression were adjusted for depression, stress, eating disorders, and diet. All *p* values were corrected with the Benjamini‒Hochberg method.

## Results

### Descriptive statistics

Each NVP severity group contained 337 unique participants. Table 1 shows the participant-level variables aggregated by group. Age and BMI were very similar between the groups, at 32 y, with a BMI of 24.6. The mean poor diet score, stress score, and depression score were greater in the high-severity group (diet *p* = 0.028, stress *p* = 0.0002, and depression *p* < 0.0001). The depression score, stress score, and nausea score correspond to the results of the EPDS, PSS-4, and PUQE questionnaires, respectively. A higher frequency of consumption of fiber-rich foods and sweetened drinks alongside lower consumption of fruits and vegetables led to higher poor diet scores. It is also more common in the high and medium groups to have had an eating disorder (*p* = 0.005).

**Table 1.** Participant-level variables in three groups of low, medium, and high NVP severity. *P*-values are calculated with Kruskal–Wallis. Variables with significant differences are highlighted in bold.

	Mean (SD)			
Variable	Low (*n* = 337)	Medium (*n* = 337)	High (*n* = 337)	*P*-value
PUQE	3.45 (0.50)	5.40 (0.49)	8.74 (1.79)	**<0.0001**
Age (y)	32.04 (4.08)	32.09 (4.29)	31.93 (4.41)	0.92
BMI	24.4 (4.63)	24.8 (4.96)	24.73 (4.99)	0.80
Gestational week	11.82 (1.86)	11.91 (2.03)	11.72 (1.93)	0.50
Depression score	6.14 (4.37)	6.57 (4.14)	7.70 (4.68)	**<0.0001**
Poor diet score	1.75 (1.14)	1.68 (1.09)	1.93 (1.16)	**0.028**
Stress score	5.02 (2.72)	5.23 (2.7)	5.97 (2.96)	**0.0002**
Smoking score	0.39 (0.50)	0.39 (0.50)	0.34 (0.51)	0.25
Socioeconomic score	2.54 (0.68)	2.55 (0.64)	2.46 (0.71)	0.23
	***N (%)***			
Current PPI usage				**<0.0001**
Yes	10 (3.0%)	15 (4.5%)	34 (10.1%)	
No	327 (97.0%)	322 (95.5%)	303 (89.9%)	
Current H1RA usage				**<0.0001**
Yes	3 (0.9%)	11 (3.3%)	34 (10.1%)	
No	334 (99.1%)	326 (96.7%)	303 (89.9%)	
Current H2RA usage				0.19
Yes	4 (1.2%)	2 (0.6%)	3 (0.9%)	
No	333 (98.8%)	335 (99.4%)	334 (99.1%)	
Primipara				0.22
Yes	154 (45.7%)	132 (39.2%)	140 (41.5%)	
No	183 (54.3%)	205 (60.8%)	197 (58.5%)	
PCOS				0.35
Yes	52 (15.4%)	65 (19.3%)	63 (18.7%)	
No	282 (83.7%)	267 (79.2%)	270 (80.1%)	
Missing	3 (0.9%)	5 (1.5%)	4 (1.2%)	
Eating disorder				**0.005**
Yes	17 (5.0%)	34 (10.1%)	41 (12.2%)	
No	313 (**92.9%**)	297 (88.1%))	293 (86.9%)	
Missing	7 (2.1%)	6 (1.8%))	3 (0.9%)	
Iron				0.29
Yes	145 (43.0%)	164 (48.7%)	146 (43.3%)	
No	189 (56.1%)	173 (51.3%)	190 (56.4%)	
Missing	3 (0.9%)	-	1 (0.3%)	
Diet group				0.74
Vegetarian	13 (3.9%)	21 (6.2%)	18 (5.3%)	
Pescetarian	28 (8.3%)	27 (8.0%)	28 (8.3%)	
Other	295 (87.5%)	289 (85.8%)	290 (86.1%)	
Missing	1 (0.3%)	-	1 (0.3%)	
Bristol rating				0.20
Slow	95 (28.2%)	100 (29.7%)	89 (26.4%)	
Normal	72 (21.4%)	55 (16.3%)	52 (15.4%)	
Fast	91 (27.0%)	84 (24.9%)	94 (27.9%)	
Various	78 (23.1%)	98 (29.1%)	101 (30.0%)	
Missing	1 (0.3%)	-	1 (0.3%)	
Sex of fetus				0.12
Boy	153 (45.4%)	156 (46.3%)	135 (40.1%)	
Girl	136 (40.4%)	134 (39.8%)	158 (46.9%)	
Missing	48 (14.2%)	47 (13.9%)	44 (13.1%)	

PPI = proton-pump inhibitor; H1RA = histamine H1 receptor antagonist; H2RA = histamine H2 receptor antagonist.

There was no difference in smoking and socioeconomic status between the groups. There is a higher percentage of baby girls in the high severity group, but it is not statistically significant (*p* = 0.12). There are slightly fewer with PCOS in the low severity group, and it is slightly more common to take iron supplements in the medium group, but these are also not significant differences.

### NVP severity correlates with depression, stress, diet quality, and eating disorder

**Figure 1.**
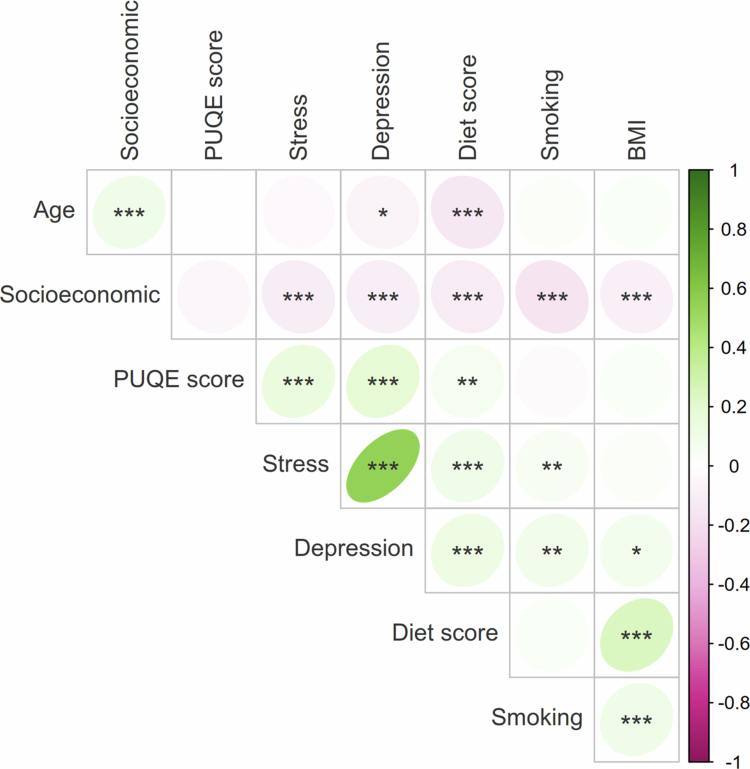
A correlation plot for continuous variables calculated with Spearman's correlation. Each value and circle represent the correlation between the variable on the x-axis and the variable on the y-axis at the intersection. The color and numbers indicate the direction of the correlation where the green spectrum and positive numbers correspond to a positive correlation, and the pink spectrum and negative numbers correspond to a negative correlation. The intensity of the color and ellipse indicates the strength and direction of the correlation. *p < 0.05; **p < 0.01; ***p < 0.001. Stress: PSS-4; Depression: EPDS.

The correlations between both NVP severity, and PUQE score and other variables were analyzed to find potential confounders or covariables for further analysis. PUQE has weak positive correlations to poor diet score (*r* = 0.07, *p* = 0.0046), depression score (*r* = 0.18, *p* < 0.0001), and stress score (*r* = 0.14, *p* < 0.0001) (Figure 1**)**. Additionally, participants with a history of eating disorders presented a significant difference in their PUQE score (Wilcoxon rank-sum test, *p* = 0.0014). The Bristol rating has a correlation to PUQE score (unadjusted *p* = 0.043) that does not hold for multiple testing correction.

Other correlations between variables were also evident in Spearman's correlation (Figure 1), such as a direct correlation between depression and the stress score (*p* < 0.0001), and with both of these and smoking (depression *p* = 0.004, stress *p* = 0.009), and unhealthy diet (t tests: depression *p* = 0.0002, stress *p* < 0.0001). Individuals who score high for depression are often younger (*p* = 0.032), have a higher BMI (*p* = 0.026) and lower socioeconomic status (depression *p* < 0.0001). An unhealthy diet also correlates with high BMI (*p* < 0.0001), lower age (*p* < 0.0001), and low socioeconomic status (*p* < 0.0001).

### NVP correlates with stool bacterial richness, but not evenness

**Figure 2.**
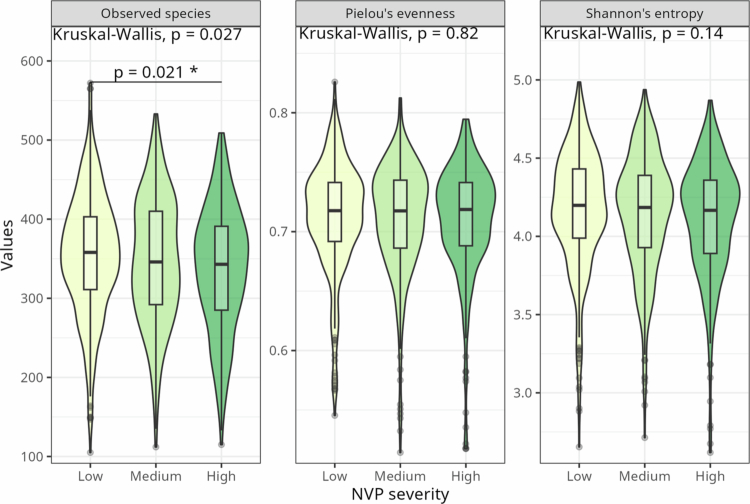
Alpha-diversity indices in NVP severity. Observed species, Pielou's evenness and Shannon's entropy as alpha-diversity in pregnant people experiencing different levels of NVP severity (measured by PUQE score divided into three groups). *P*-values were calculated with the Kruskal‒Wallis test and adjusted for multiple testing within each alpha index with the Benjamini‒Hochberg method. Adjusted, significant *p*-values are displayed for pairwise comparisons.

Richness, evenness, and Shannon's entropy were compared between NVP severity groups, showing decreasing richness with increasing nausea (Kruskal‒Wallis *p* = 0.027, adjusted Wilcoxon's post-hoc high vs. low *p* = 0.021; Figure 2).

The PUQE score correlates negatively with Shannon's entropy (*p* = 0.0043) and observed species (*p* = 0.0002) but not with Pielou's evenness (*p* = 0.12) (Figure S1). Observed species number also correlates negatively to poor diet score (*p* = 0.0035), stress score (*p* = 0.0012), and depression score (*p* = 0.0014). A history of eating disorder was not connected to any significant differences in alpha-diversity (Welch's t-test: Shannon's entropy, *p* = 0.3; Pielou's evenness, *p* = 0.07; observed species richness, *p* = 0.9).

### Association between NVP and beta-diversity

Jaccard and Aitchison distances were used to assess whether there is any difference in beta-diversity between NVP severity groups. PERMANOVA was used to find statistical differences, first as a univariable model for each variable: PUQE score, NVP severity, stress, depression, diet, and eating disorder and then as multivariable models. In the univariable model, the beta-diversity was significantly different in both Aitchison distance and Jaccard dissimilarity for all variables except eating disorder and NVP severity (Figure S2). Eating disorder was not significant for either Aitchison or Jaccard, and NVP severity was only significant in Jaccard.

**Figure 3.**
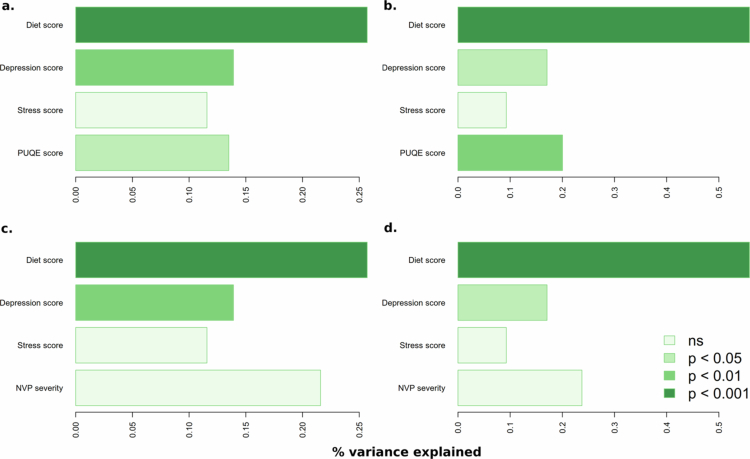
Multivariable PERMANOVA with adonis2. The *p*-values (color) and variance (x-axis) for different multivariable models where the order from top to bottom for each figure is the order of variables used in the model. The left column of figures displays models built with Jaccard dissimilarity used as the outcome variable and the right column displays the results with Aitchison distance used as the outcome variable.

The significant variables from the univariable analyzes were used to build a multivariable model for each distance metric with PUQE score and NVP severity in either the first or the last position, since the Permanova function fits variance onto variables consecutively. PUQE score remains significant in both the first and last position, indicating that the difference in beta-diversity seen for PUQE score is not explained by either stress, depression, or poor diet score (Figure 3a-b). NVP severity on the other hand is close to significant when placed in the first position for Jaccard distance but not for Aitchison distance, and can be better explained by a combination of diet and depression scores (Figure 3c-d). As depression score has a higher explanatory value than stress score, stress score was not included in the model going forward.

In the final multivariable model, PUQE score can explain roughly 0.25% of the variance in the Jaccard model and 0.15% in the Aitchison model. Diet explains roughly 0.5% of the variance in the Jaccard model and half of that (~0.25%) in the Aitchison model. Depression explains roughly 0.2% of the variance in the Jaccard model and 0.13% percent of the variance in the Aitchison model.

Taken together, these results show a significant difference in beta-diversity measured as both Jaccard dissimilarity and Aitchison distance based on the PUQE score, but there is no significant difference between NVP severity groups. PUQE score explains roughly 0.2% of the variance for both multivariable models of Aitchison and Jaccard. Depression score and poor diet score also significantly affected the models and explain an additional part of the variance.

### Species that are differentially abundant in relation to PUQE score

To assess which species were driving the observed differences in alpha- and beta-diversity, ANCOM-BC was run as both using only PUQE score as the explanatory variable and in a multivariable model including poor diet score and depression score as explanatory variables. In the unadjusted model, 228/773 species had a significantly different log-fold change in relative abundance (Table S5). In the adjusted model, 244 species were significant (Table S6). Out of these, 208 species (78.8%) were significant in both models. The species that are only significant in one model are close to the cutoff for both models, while those that have the largest log-fold count difference are significant in both (Figure S3).

**Figure 4.**
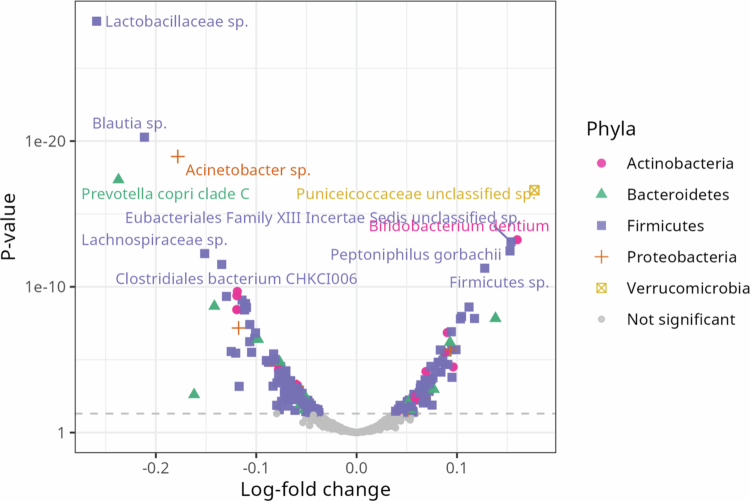
Volcano plot for adjusted differential abundance analysis, with PUQE score as the response variable. The y-axis is the *p*-values, and the x-axis is the log-fold change per unit of PUQE score. Color and shape indicate phyla. The species with the highest and lowest log-fold change has been marked with species name where that is known and the lowest known taxonomic group where species are not known.

Among the species with the highest difference in relative abundance are many unknown species (Figure 4). The relative abundance of several of these, including *Prevotella copri* clade A and clade C, and *Blautia sp*., decrease with PUQE score. The relative abundance of *Bifidobacterium dentium*, *Peptoniphilus gorbachii*, *Phocaeicola plebeius,* and several unknown species increase with PUQE score.

### NVP and gut‒brain and gut‒metabolic modules

All 148 gut–brain and gut-metabolic modules were detected in our sample. Of these, 27 were discarded due to low prevalence (<15%), 32 modules showed moderate prevalence between 15% and 85% and were analyzed as presence–absence, and 89 modules were found to have high prevalence (>85%) and were analyzed using partial Spearman correlation.

**Figure 5.**
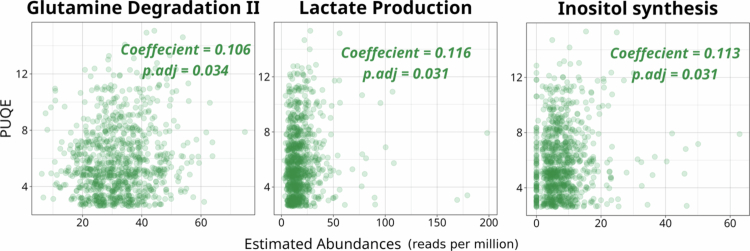
Glutamine degradation, lactate production and inositol synthesis are significantly associated with the PUQE score. a) Glutamine degradation II, b) lactate production, and c) inositol synthesis were positively correlated with PUQE scores after BH *p* value adjustment. The x-axis shows the estimated abundance of the modules and the y-axis represents the PUQE score. The space between sample points along the x axis were randomly increased for better data visualization.

None of the modules with moderate presence were associated with the PUQE score past multiple-testing correction (all adjusted *p* > 0.5). Out of the 89 highly prevalent modules, three were found to be significantly correlated with PUQE score after multiple testing correction, namely glutamine degradation II (adjusted *p* value = 0.034), lactate production (adjusted *p* value = 0.031), and inositol synthesis (adjusted *p* value = 0.031). All three were found to be positively correlated with PUQE scores (Figure 5). Additionally, *Blautia sp*., which was found to be associated with decreased PUQE score, was among the top five most abundant species carrying the glutaminase (K00265) gene *gltB* in the glutamine degradation II pathway.

## Discussion

Here, we present a thorough analysis of the gut microbiome around week 12 of pregnancy, in relation to nausea and vomiting. We have included a large amount of participant-level variables including medication, diet, and medical history. We have used various metrics both for quantifying the severity of NVP (groups vs. PUQE) but also for quantifying alpha- and beta-diversity. These led to slight differences in results, but with an overall good agreement that points towards richness, or the absence of certain species, as the main driving factor for the association between NVP and the gut microbiome. Previous studies have found a decrease in gut microbiome richness in late pregnancy,^49^ and symptoms of NVP also decrease later in pregnancy.^50^ As such it would be counterintuitive that richness is lower in those with higher severity of NVP. Perhaps there is a difference in which species are decreased normally in pregnancy, and which species are lacking in severe NVP. It is also possible that, much like many physiological parameters change over the course of a healthy pregnancy, the microbiota also needs to transform at a proper pace, with a premature decrease in richness signifying something else than the physiological decrease observed in late pregnancy.

Depression and anxiety have previously been found to correlate with HG,^51^ which we also found, despite using different psychometric scales and considering the outcome as NVP, rather than HG. Despite this correlation, PERMANOVA models showed significant correlation between fecal microbiome and PUQE score, even when first fitting on stress or depression. Additionally, persons with eating disorders have been shown to be more likely to experience HG,^52^ and also to have a different gut microbiota composition.^53^ Interestingly, Teräväa-Utti and colleagues found a bidirectional correlation between eating disorders and HG, with women with a previous eating disorder being more likely to experience HG, but also women with HG being at higher risk of a new diagnosis of eating disorder. This suggests that there may be common underlying risk factors to both diagnoses.

Previous research shows that, among other foods, a decrease in fruits and increase in soft drinks is associated with higher levels of NVP.^54^ The poor diet score in this study was based on frequency of consumption of fiber-rich foods and sweet drinks, such that less consumption of fruits and vegetables and more consumption of sugary or sweetened drinks lead to a higher score. We also observed a positive correlation between the poor diet and PUQE scores, which matches the earlier findings well. What is harder to disentangle is whether more severe NVP leads to this change in diet, or whether the change in diet leads to higher levels of NVP, especially considering the causative link between diet and microbiota,^55^^,^^56^ but also between nausea and diet.^54^ Diet score correlated to diversity metrics, and in the multivariable model, diet and depression together explained some of the variance in PUQE score. More studies into the interplay between pregnancy, microbiome, diet and nausea are needed, as the current evidence offers little in terms of specific food items to alleviate nausea and vomiting.^57^

More than 200 species differed in relative abundance for those with severe NVP and controls, many of which have never been isolated. *Bifidobacterium dentium*, which converts glutamate to GABA and has previously been implicated in reduced visceral hypersensitivity,^58^ was one of the species with the clearest positive correlation to PUQE score. On the other hand, participants with severe NVP displayed a decreased relative abundance of *Prevotella copri*, whose beta-glucuronidases are known to modulate active estrogen concentrations in circulation, by making estrogens available for reabsorption in the gut.^59^

In addition to these species, three metabolic pathways were increased in subjects with higher PUQE score, of which two have known neuroactive potential, namely inositol synthesis and glutamine degradation,^42^ with glutamine serving as an obligatory precursor of glutamate and GABA. Inositol phospholipids are key precursors to G-protein coupled receptors and have been considered for the treatment of various psychiatric disorders, with the most promising results being found for panic disorder and obsessive–compulsive disorder.^60^ However, it has also been noted in trials that high doses of inositol lead to nausea as a side effect.^61^ Despite several clinical trials in a wide range of psychiatric conditions, effects remain unclear and the use of inositol outside of clinical trials is not currently recommended.^62^ Glutamate has well-known neurophysiological effects, although its implication in mood disorders are still debatable.^63^ The consumption of monosodium glutamate (MSG) has been linked to higher risk of nausea,^64^ possibly due to the activation of excitatory neurons in the area postrema of the brain stem (potentially leading to emesis).^65^ On the other hand, MSG is a common food additive of ultraprocessed foods,^66^ so that this observation could be a simple effect of lower diet quality. Interestingly, *Blautia* sp., which was associated with reduced PUQE scores, ranked among the top five most abundant species harboring the glutaminase gene (*gltB*) involved in the glutamine degradation II pathway. *Blautia* species have been increasingly recognized for their probiotic potential^67^ and their capacity to maintain intestinal homeostasis through metabolite production and anti-inflammatory effects. Our findings raise the possibility that *Blautia* sp. may influence host neurophysiology by modulating glutamine degradation and glutamate synthesis, thereby attenuating excitatory signaling^68^ and mitigating nausea symptoms. However, as these functional inferences are derived from shotgun metagenomic annotations rather than transcriptomic or metabolomic measurement, the contribution of *Blautia*-mediated glutamine metabolism to host neurotransmission remains to be further validated.

The cross-sectional nature of this study limits causal inference. Information on nausea, diet, and medication usage were collected simultaneously, typically 1–2 weeks prior to stool sample collection. There is therefore a risk of reverse causation, in that nausea itself leads to behavioral changes (diet, medication) and alters the stool microbiome composition, especially considering the large impact of PPI on the stool microbiome.^69^ However, given the difficulties of recruiting pregnant women before the typical peak of nausea during weeks 8–12, we believe that this paper presents an important contribution to understanding the possible connection between the gut microbiome and NVP.

Another weakness of this study is that, while the gut microbiome is fairly stable, NVP symptom profiles have a lot of individual variation^2^; therefore, there could be participants that report a low PUQE score at the time of sampling, but nevertheless suffered with higher levels of NVP earlier or later in their pregnancy. We partially alleviated this issue by matching participants based on their pregnancy week at sampling.

In summary, after a thorough analysis of NVP in relation to species- and functional composition of the gut microbiome, we found that pregnant women with high NVP had fewer species in their guts, but an increase on relative abundance of over a hundred species, of which the most extreme are *Bifidobacterium dentium, Peptoniphilus gorbachi, Phocaceicola plebeius,* and uncultured members of the families *Puniceicoccaceae* and Eubacteriales family XIII. Additionally, there was an indication that high NVP subjects were exposed to higher levels of glutamate, which could partially explain their symptoms. This suggests further possibilities to ameliorate symptoms of early pregnancy through pharmacological or dietary interventions.

## Supplementary Material

Supplementary material Supplementary Material.

Supplementary material Supplementary Material.

Supplementary material Supplementary Material.

Supplementary material Supplementary Material.

## Data Availability

Microbiome data, as well as limited participant data, are available on the European Nucleotide Archive (ENA): https://www.ebi.ac.uk/ena/browser/view/PRJEB81814. Additional data on participants may be provided by reasonable request, provided that it does not risk the anonymity of our volunteers or otherwise constitute a breach of ethics.
