# Supplementary figures and images for: The gut microbiome in early pregnancy is associated with the severity of nausea and vomiting: a nested case‒control study
Source: Gut Microbes Rep. 2025 Dec 22;3(1):2603861. doi: 10.1080/29933935.2025.2603861 (PMC12938880; doi:10.1080/29933935.2025.2603861)

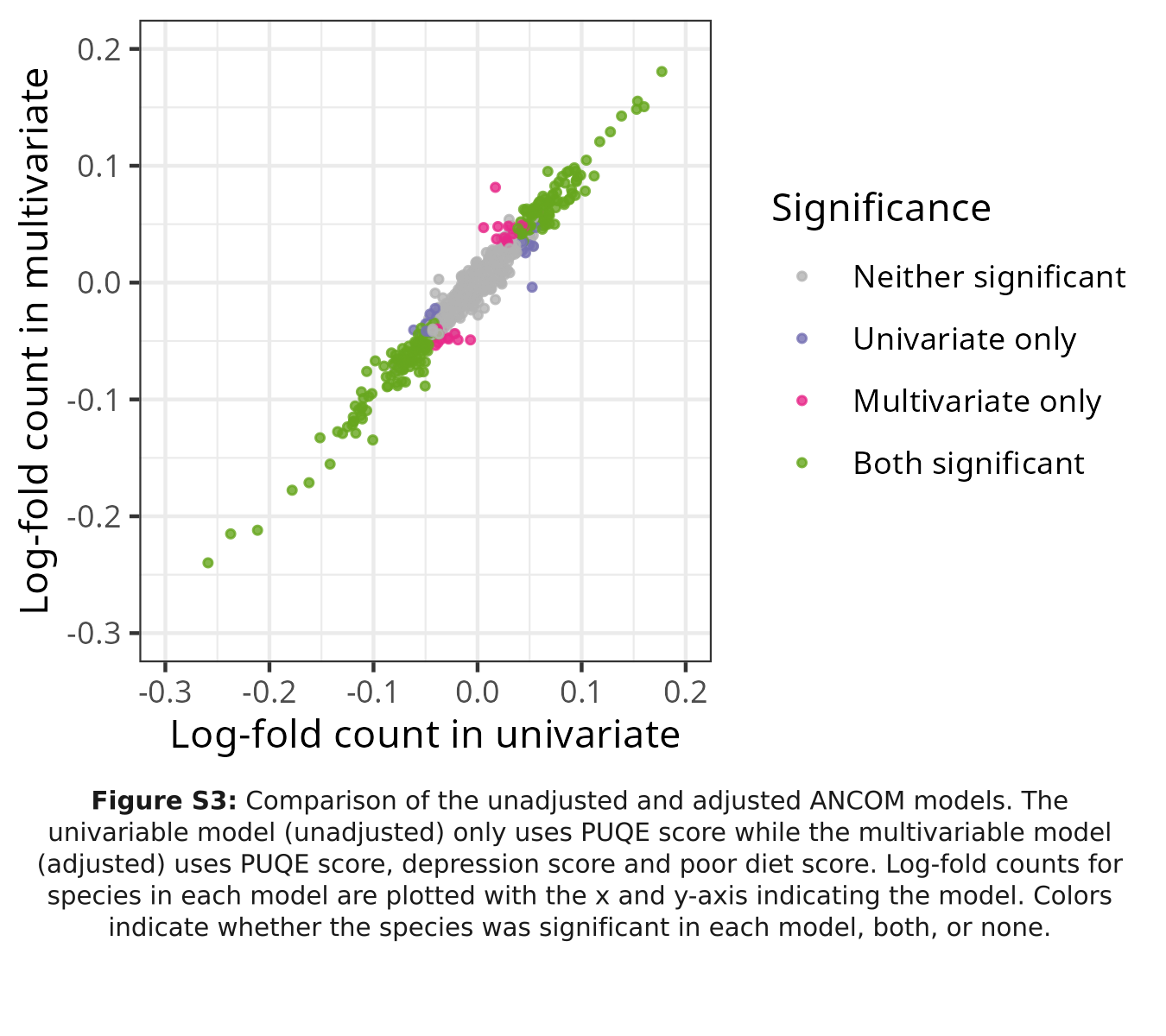

Supplement: Supplementary material [file KGMR_A_2603861_SM2160.tif]

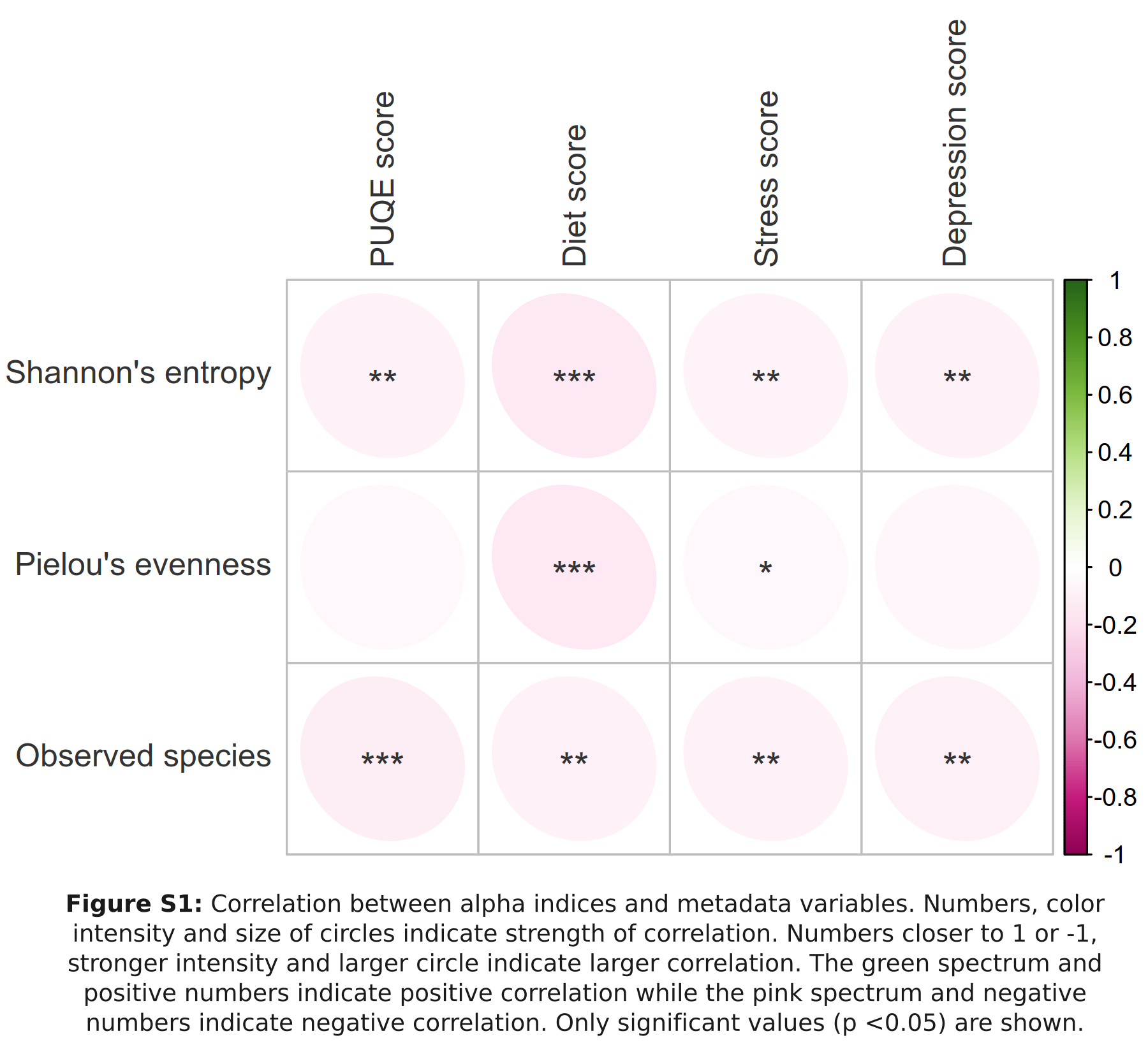

Supplement: Supplementary material [file KGMR_A_2603861_SM2161.tiff]

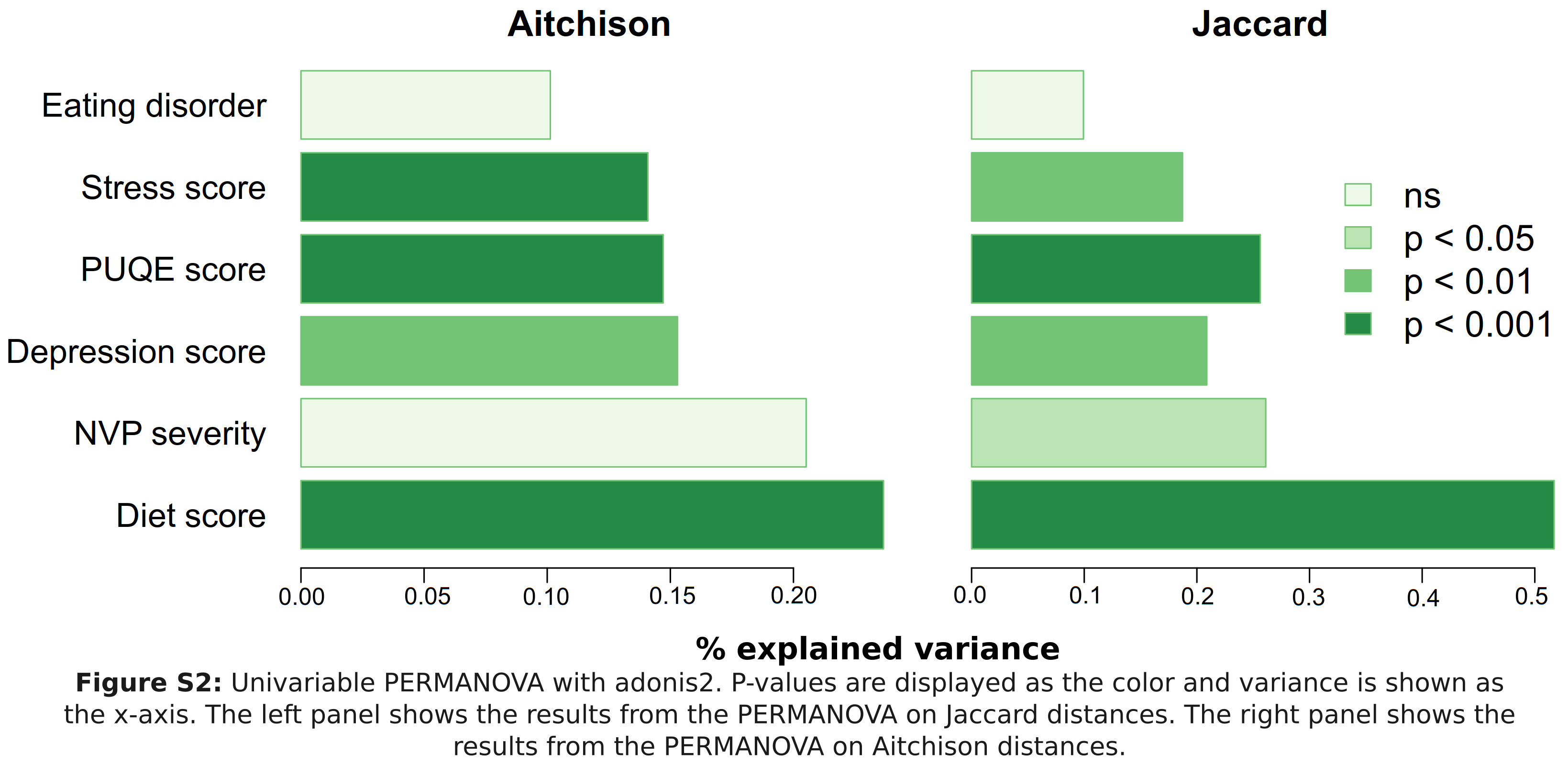

Supplement: Supplementary material [file KGMR_A_2603861_SM2162.tif]
